# Positive affective priming decreases the middle late positive potential response to negative images

**DOI:** 10.1002/brb3.1198

**Published:** 2018-12-19

**Authors:** Lauren D. Hill, Valerie G. Starratt, Mercedes Fernandez, Jaime L. Tartar

**Affiliations:** ^1^ Department of Psychology & Neuroscience Nova Southeastern University Ft. Lauderdale Florida

**Keywords:** attention, emotion, ERP, foveal, LPP, priming

## Abstract

**Introduction:**

This study aims to expand on previous literature showing that incidental emotion state priming in a specific domain leads to a higher probability that the primed emotion domain will be activated during a subsequent task.

**Methods:**

To that end, we investigated the influence of happy, fearful, and neutral incidental emotion state priming on subsequent responses to emotionally negative and neutral pictures, measured by the event‐related potential (ERP) late positive potential (LPP). New to our study, we examined the influence of affective priming on the LPP response (analyzed separately at early and middle latency ranges) to emotional pictures in both the foveal and extrafoveal presentation locations.

**Results:**

Following both fearful and neutral incidental state priming, both the early and middle LPP latency ranges overwhelmingly differentiated between negative and neutral pictures. Following happy incidental state priming, however, the LPP response failed to differentiate between negative and neutral pictures by the middle LPP latency range (800–1,000 ms). These results suggest that incidental happy states can have a protective effect when viewing aversive stimuli. Additionally, the LPP showed greater sensitivity to negative stimuli when presented extrafoveally compared to foveally.

**Conclusions:**

Overall, our findings suggest that incidental affective state and stimulus location influence emotional processing differentially for emotionally negative and emotionally neutral stimuli.

## 1 INTRODUCTION

Emotions function as superordinate neurocognitive programs that organize and prioritize a person's perception of and response to the world in a way that is likely to have adaptive value (Cosmides & Tooby, 2000; Ekman, 1992). These adaptive values can be grossly divided into two functional motivational systems: those that motivate a person to move toward stimuli that could provide some benefit, and those that motivate a person to move away from stimuli that indicate danger. The translation of emotion to a potentially beneficial response occurs via activation of one of the two motivational systems (Lang, Bradley, & Cuthbert, 1997a, 1997b).

The first of these systems, the appetitive system, translates positive emotions into behavior that moves a person *toward* a potentially rewarding experience. For example, the perception of highly arousing, positive pictures (e.g., erotica) produces an increase in attention to those pictures, as evidenced by both functional magnetic resonance imaging (fMRI; Bradley et al., 2003; Lang et al., 1998) and electrophysiological recording (Olofsson, Nordin, Sequeira, & Polich, 2008; Schupp et al., 2004). Like positive emotions, negative emotions also increase attention to relevant (which in this case means negative) stimuli (Bradley et al., 2003; Schupp et al., 2004). Unlike positive emotions, though, this increased activation represents the second aspect of motivation, the defensive motivational system. Negative emotions are more likely to be associated with punishing than rewarding experiences, and so movement toward the source of a negative emotion could be considered ill‐advised. Rather, moving *away* from a negative stimulus would likely be the safer option, which is the function of the defensive motivation system (Rinck & Becker, 2007).

Particularly relevant to the present study is the idea that motivational affective signals can moderate neural processing (Aarts, Custers, & Veltkamp, 2008). The ability of neural processing resources to extend in time to influence the processing of subsequent stimuli is thought of as “affective” or “motivational” priming and is most reliably demonstrated when priming occurs implicitly, outside of conscious awareness (Bargh, Gollwitzer, Lee‐Chai, Barndollar, & Trötschel, 2001; Bargh & Williams, 2007). For example, using EEG event‐related potentials (ERPs), we have previously shown that the induction of emotionally negative states—through stress or sleep deprivation—results in the inability of neural processing to discriminate between neutral and affectively laden visual stimuli (Alfarra, Fins, Chayo, & Tartar, 2015; Alomari, Fernandez, Banks, Acosta, & Tartar, 2015). Similarly, we have shown that visual affective priming can moderate neural responses to subsequent stimuli across sensory domains, as evidenced by increases in ERP measures of attention to rarely occurring auditory stimuli (Tartar, de Almeida, McIntosh, Rosselli, & Nash, 2012).

Such relationship between incidental state emotion and attention to emotional stimuli likely results from shared limbic processing networks. For example, incidental priming with emotionally laden words is associated with increased activation of emotion networks in the orbitofrontal gyrus and bilateral inferior frontal gyrus (Kuchinke et al., 2005). Similarly, incidental affective priming with a sad video clip produces greater amygdala activation in response to sad pictures relative to happy and neutral pictures (Wang, LaBar, & McCarthy, 2006). In general, then, it seems incidental emotional state priming in one emotional domain results in a higher probability that the corresponding motivational system will be subsequently activated.

In the current study, we aimed to expand contemporary literature on the relationship between affective priming and subsequent motivated attentional responses in two ways, through assessing both the affective category of the prime and the affective category of the stimuli. First, we investigated the influence of two incidental emotional states—happy and fearful, as induced via presentation of affectively laden videos—on the late positive potential (LPP) ERP response to subsequent negative and neutral visual stimuli. We chose emotionally negative stimuli as the target because, compared to appetitive (or pleasant) stimuli, emotionally negative (or unpleasant) stimuli typically produce stronger emotional responses (Crawford & Cacioppo, 2002; Öhman & Mineka, 2001; Schupp et al., 2004; Smith, Cacioppo, Larsen, & Chartrand, 2003).

As in our previous work, we chose the LPP ERP as our primary dependent measure of emotion processing because the LPP has repeatedly been observed in response to arousing stimuli of both positive and negative valence (Cuthbert, Schupp, Bradley, Birbaumer, & Lang, 2000; Hajcak & Olvet, 2008; Palomba, Angrilli, & Mini, 1997), but not to affectively neutral stimuli. The LPP is a reliable index of motivated attentional processing, particularly as it relates to picture stimuli (Ferrari, Codispoti, Cardinale, & Bradley, 2008; Gable & Poole, 2014; Olofsson et al., 2008). Attention is a multifaced concept that has been defined as information processing that involves *both* processes of selection and evaluation of motivationally relevant input in order to respond to environmental stimuli (Lang, Bradley, & Cuthbert, 1997a, 1997b). This suggests an interplay between mechanisms involved in behavioral response (motivation) and emotional evaluation (processing). The LPP has been shown to be associated with behavioral motivation where withdrawal motivation systems modulate reactions to aversive stimuli, and approach motivation systems modulate reactions to appetitive or rewarding stimuli (Gable & Harmon‐Jones, 2010a, 2010b). Of note, unlike other ERP components, the LPP does not habituate over repeated stimuli presentation and is stable over time within an individual. This indicates that the LPP is driven by motivational salience, and not by stimulus novelty or violations of expectation (Hajcak, MacNamara, & Olvet, 2010).

Further, the time course and topology of the LPP have shown to provide critical insight into affective processing, where the LPP tracks an individual's responsiveness to emotional material over time. In fact, previous research has shown a spatial–temporal shift pattern that the LPP was maximal at posterior–superior recording sites in the early window (300–600 ms), but shifted to posterior and anterior recording sites during the middle (600 to 1,000 ms) and late windows (1,000–2000 ms) during emotional reappraisal (Dennis & Hajcak, 2009; Hajcak et al., 2010; Hajcak & Nieuwenhuis, 2006) and emotional modulation (Moser, Hajcak, Bukay, & Simons, 2006). Exploring the time course of the LPP during altered incidental state emotion has potential to provide insight into the temporal mechanisms underlying sustained attention to emotional stimuli during varied emotional states. Given these characteristics, the LPP ERP component is commonly used as an index of emotional processing, with experimental evidence supporting the theory that motivated attention is driven by both the appetitive and aversive motivational systems (Wiens & Syrjänen, 2013).

Regarding the first aim of the current study, we specifically predicted that the negative affective priming would activate the aversive motivational networks and result in an LPP ERP response that was increasingly sensitive to negative pictures. We further predicted that, relative to the neutral video condition, happy affective priming would activate the appetitive network and result in a reduced LPP ERP response to negative pictures.

The second aim of this study was to investigate whether the relationship between affective priming and attentional motivational systems could be moderated by stimulus presentation location. In general, the ability to quickly detect potential threats in the environment is essential for survival, and hence, *where* the information appears in the visual field (i.e., the center of the visual field vs. elsewhere in the visual field) is critical information. Indeed, visual structures afferented by the extrafoveal retina process emotionally relevant and salient cues (Bayle, Schoendorff, Hénaff, & Krolak‐Salmon, 2011). The effective detection of extrafoveal negative stimuli could be particularly advantageous, as such an image indicates not just potential danger, but specifically potential danger that has not yet been brought to the center of attention for an assessment of its true threat value. Consequently, negative stimuli perceived extrafoveally may represent the kind of stimulus most likely to activate aversive motivated attention. For that reason, and new to our study, we predicted that the LPP response to negative stimuli following affective priming would be particularly pronounced for stimuli presented extrafoveally.

In sum, the aims of the current study were to (a) expand the investigation into the relationship between incidental emotional state and motivated attention, assessed via the LPP ERP component, by manipulating emotional state directly (via the presentation of affectively laden videos) rather than indirectly (e.g., via acute stress or sleep deprivation) and to (b) determine the extent to which this relationship might be moderated by stimulus presentation location.

## 2 METHODS

### 2.1 Participants

Fifty‐three undergraduate students were recruited for participation in exchange for partial course credit. A demographics questionnaire assessed participants’ age, gender, race, handedness (right or left), ethnicity, medications, and family history of mental illness. Individuals who were left‐handed or currently taking medications that could have altered their EEG recordings were excluded from participation. All participants were right‐handed, reported no depressive or anxious episodes within the last 6 months, reported taking no medication (other than birth control or ibuprofen), and were between the ages of 18 and 30 years old with normal hearing and normal or corrected‐to‐normal vision. Five participants had excessive movement and/or eye artifact during the recording with a low number (<20) of artifact‐free trials. The amplitudes of the LPPs generated from these participants were found to be outliers, with mean ERP amplitudes greater than three standard deviations from the sample mean. Accordingly, these participants were removed from all additional analyses, leaving a total sample of 49 participants across the three conditions (18 neutral control, 15 happy, 16 fear), with a mean age of 19 years (*SD* = 3.7). All participants signed an informed consent prior to study participation, and experimental procedures were carried out according to the protocol approved by the Nova Southeastern University Institutional Review Board (IRB).

### 2.2 Visual stimuli

#### 2.2.1 Videos

As with previous work, we used validated video clips to induce an implicit emotion or motivation state for affective priming (Gabert‐Quillen, Bartolini, Abravanel, & Sanislow, 2015; Gross & Levenson, 1995). Video induction of implicit emotion was used in the current study to induce a “fear,” “happy,” or neutral emotion. Participants were randomly assigned to watch one of three videos, each of which had been validated to induce the desired emotional state.

#### 2.2.2 Happy

Participants assigned to the happy condition watched a clip from the movie Wall‐E (2008). The clip begins (58:51) with a white robot flying forward. Two robots fall in love and dance in outer space as people in a spaceship watch and music plays. The clip ends when the two robots fly away together, before the shot of a spaceship (1:02:06). The total time of the clip is 3 min and 15 s (Gabert‐Quillen et al., 2015).

#### 2.2.3 Fear

Participants assigned to the fear condition watched a clip from the movie The Ring (2002). The clip begins (1:39:28) with a man working. The TV then turns itself on, and a girl begins to crawl out of the TV and pulls her hair out of her face. The scene is interspersed with frames where the girl is trying to reach out to the man. The clip ends on static (1:42:13). The total time of the clip is 2 min and 45 s (Gabert‐Quillen et al., 2015).

#### 2.2.4 Neutral

Participants assigned to the affectively neutral condition watched a clip from Alaska's Wild Denali (1997), which served as a nonemotional video control. The clip begins (33:15) right after a person plays a guitar. Music is playing, and the visual silhouette of a mountain appears. The clip is interspersed with frames of animals, people, and nature. The clip ends (38:30) as a buck is eating grass and mountains appear. The total time of the clip is 5 min and 2 s (Gross & Levenson, 1995); however, it was shortened to 2 min and 16 s to be consistent with the other two films.

#### 2.2.5 Pictures

Forty‐five negative and 45 neutral color pictures were selected from the 2008 IAPS database (Lang, Bradley, & Cuthbert, 2008). Each image was presented twice, once foveally (0º from a central fixation point) and once extrafoveally (20º to the right or left of a central fixation point). The IAPS normative ratings were used to select the emotional category of each picture (Lang et al., 2008). The average normative rating was as follows: negative valence = 2.35, negative arousal = 5.71, neutral valence = 5.22, neutral arousal = 3.80. As a manipulation control, participants were asked to report a valence rating for each picture during the foveal trials. Participants responded on a computer keypad during the task, and the responses were collected in the Curry software.

#### 2.2.6 Visual analog scale

The visual analog scale (VAS) was used as a manipulation check for the induction of mood changes. Immediately after the video presentation, participants were instructed to mark with an “X” on a horizontal 100 mm line how they were currently feeling. The line was anchored with the descriptors “negative” at 0 mm, “neutral” at 50 mm, and “positive” at 100 mm. The VAS score was recorded as the distance (in mm) from zero to where the participant marked an “X” through the line.

### 2.3 Procedure

Upon arrival at the scheduled time and location, all participants completed a demographics form, were comfortably seated and fitted with an electrode cap and EOG electrodes, and were instructed to begin the experimental task. The task began with practice trials until the participant felt comfortable with the experimental protocol. EEG was not recorded during practice trials, which did not include any images used in the experimental trials. Following practice, participants viewed either the happy, fearful, or neutral video, and then immediately completed the VAS. Upon VAS completion, participants were instructed that experimental trials were to begin, and were reminded to restrict their movements to avoid interference with the EEG recording.

The experimental session consisted of 180 trials, including 45 neutral and 45 negative images, each of which was presented twice (once foveally and once extrafoveally). The images were randomized and presented for 2000 ms, with a 2000 ms interstimulus interval, using Stim2 software (RRID:SCR_016751, Compumedics USA Inc., Charlotte). For images presented in the center of the screen (foveal), participants were instructed to use the keyboard to rate the valence of the image on a scale of 1 (negative) to 9 (positive) during the inter‐stimulus interval. To ensure extrafoveal processing of images presented in the left and right fields of view (off center), participants were instructed to keep their focus on the fixation cross shown in the center of the monitor screen. No keyboard response was requested for stimuli presented extrafoveally, per directed focus on the center cross. Following completion of the 180 trial experimental session, EEG recording was terminated. Participants were disconnected from the amplifier, and all EEG equipments were removed. In closure, participants were debriefed, thanked, and assured they would be receiving course credit for participation.

### 2.4 Electroencephalographic recording and data processing

Continuous EEG recordings were collected from 64 active electrodes using the Compumedics Quick‐Cap EEG 64 channel cap and Nuevo 148362 amplifier (Compumedics USA Inc., Charlotte). In addition, two mastoid reference electrodes were placed behind each ear and four facial EOG (1 cm above and 1 cm distal to each eye) recorded eye movement and blinks. Electrode impedance was maintained at <10 kΩ.

The EEG amplifier was set at a sampling rate of 1,000 Hz. The data were analyzed offline through the use of Curry 7 software (RRID:SCR_009546; Compumedics USA Inc.). All data were referenced to M1, M2, and baseline; correction/bad block removal was set to constant. High‐pass filters were set to 0.1 Hz (slope = 0.2), and low‐pass filters were set to 40 Hz (slope = 8.0). A 60 Hz notch filter (slope = 1.5) with harmonics on was selected. A semiautomatic procedure was employed to detect and reject artifacts. Trials where the EOG exceeded ±75 μV were corrected using a covariance technique. Visual inspection of the continuous data confirmed that there were no remaining artifacts. For the ERP analysis, 1,000 ms of raw EEG data was epoched to the respective stimulus presentation including a 100 ms prestimulus baseline. ERPs were organized by picture type and location (negative‐foveal, neutral‐foveal, negative‐extrafoveal, and neutral‐extrafoveal) for each of the three incidental emotion conditions (neutral control, happy, fearful).

Because the LPP ERP is maximal at centro‐parietal sites (Foti & Hajcak, 2008), it was scored as the average activity from five sites (Cz, Pz, CPz, CP1, and CP2). Previous research has demonstrated that early and later windows of the LPP may reflect differences in the time course of emotional responding (Weinberg & Hajcak, 2011), and we visually observed a change in the amplitude at approximately 800 ms. The LPP was therefore examined in two time windows: early (400–800 ms) and middle (800–1,000 ms; Weinberg, Hilgard, Bartholow, & Hajcak, 2012).

### 2.5 Statistical analyses

First, we conducted a paired samples *t* test to replicate the established effect of stimulus valence on LPP amplitude and a one‐way independent ANOVA to confirm the differential influence of the affective manipulation between groups. We then conducted a 3‐way mixed‐model ANOVA to examine the effects of condition (neutral prime, happy prime, fear prime), LPP latency range (early, middle), and stimulus presentation location (foveal, extrafoveal) on LPP amplitude difference in response to negative vs. neutral pictures. All analyses were performed in R 3.2.4.

## 3 RESULTS

### 3.1 Replication

Consistent with previous research, in our affectively neutral prime (i.e., control) condition, the LPP differentiated negative from neutrally valenced stimuli, *t*(17) = 2.61, *p* < 0.05, *d* = 0.62. LPP amplitude was significantly larger in response to negative (*M* = 5.6 µV, *SD* = 10.1) compared to neutral pictures (*M* = 0.6 µV, *SD* = 7.1).

### 3.2 VAS manipulation check

**Figure 1:**
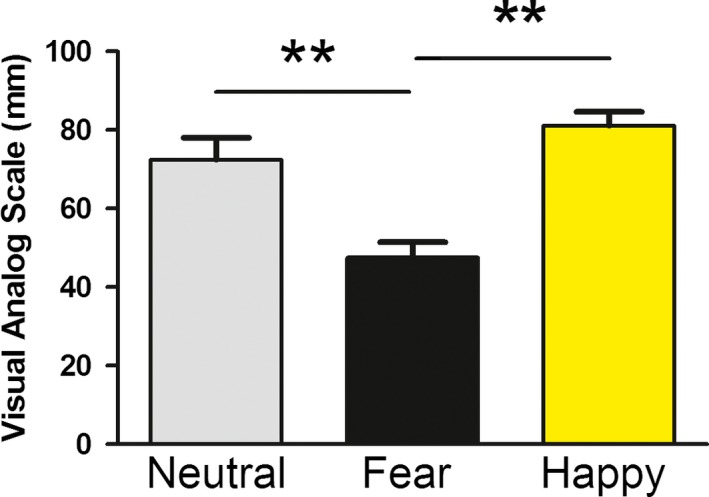
A visual analog scale was administered after the video prime as a manipulation check for the induction of mood changes. Participants were instructed to mark with an “X” on a horizontal 100 mm line how they were currently feeling ranging from negative at 0 mm to positive at 100 mm

As seen in Figure 1, a one‐way independent ANOVA confirmed significant group differences in affect between the video conditions, *F*(2, 48) = 14.05, *p* < 0.01, partial *η* ^2^ = 0.38. Follow‐up analyses revealed that, relative to the neutral condition (*M* = 72.4, *SD* = 23.6), the fear condition (*M* = 47.6, *SD* = 15.5) resulted in significantly greater negative affect (*p* < 0.01) and the happy video condition (*M* = 78.9, *SD* = 16.5) resulted in significantly greater positive affect (*p* < 0.01).

### 3.3 3‐way mixed‐model ANOVA

**Figure 2:**
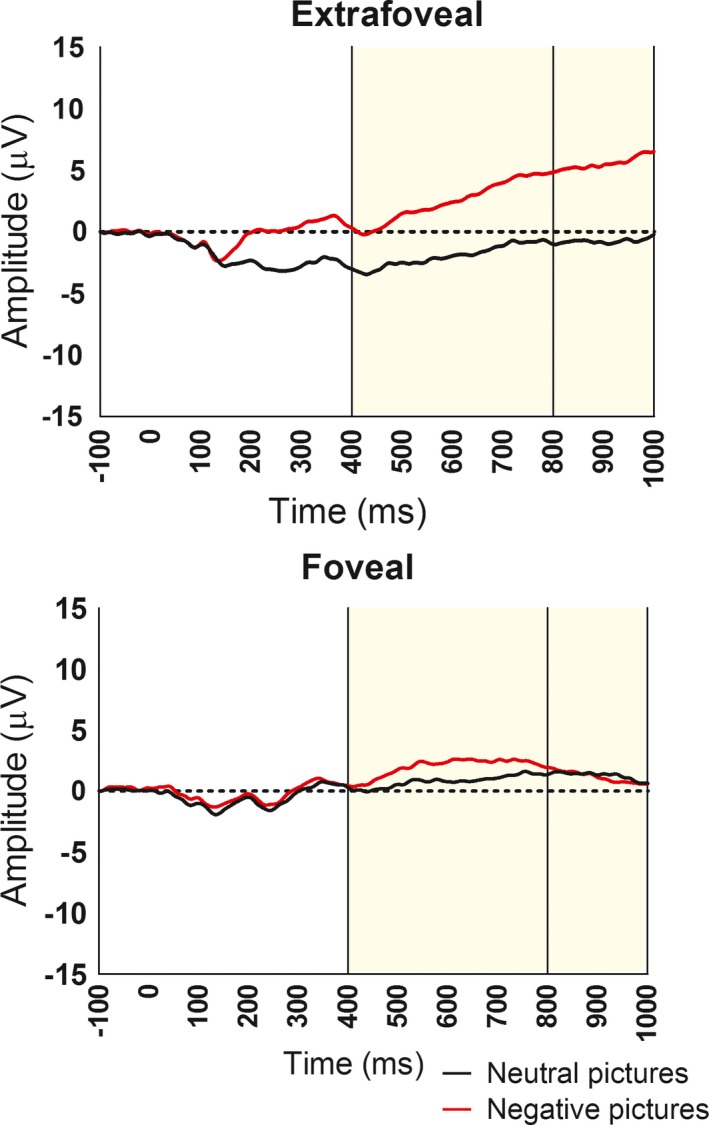
Visual LPP ERPs in response to emotionally negative (red) or emotionally neutral (black) images following a neutral (top), fearful (middle), or happy (bottom) video prime, collapsed across presentation location (foveal, extrafoveal). Participants were exposed to an image for 2000 ms. The LPP ERP was scored as the average activity from five sites (Cz, Pz, CPz, CP1, and CP2). *Y*‐axis represents voltage (µV), and *x*‐axis represents time (ms)

**Figure 3:**
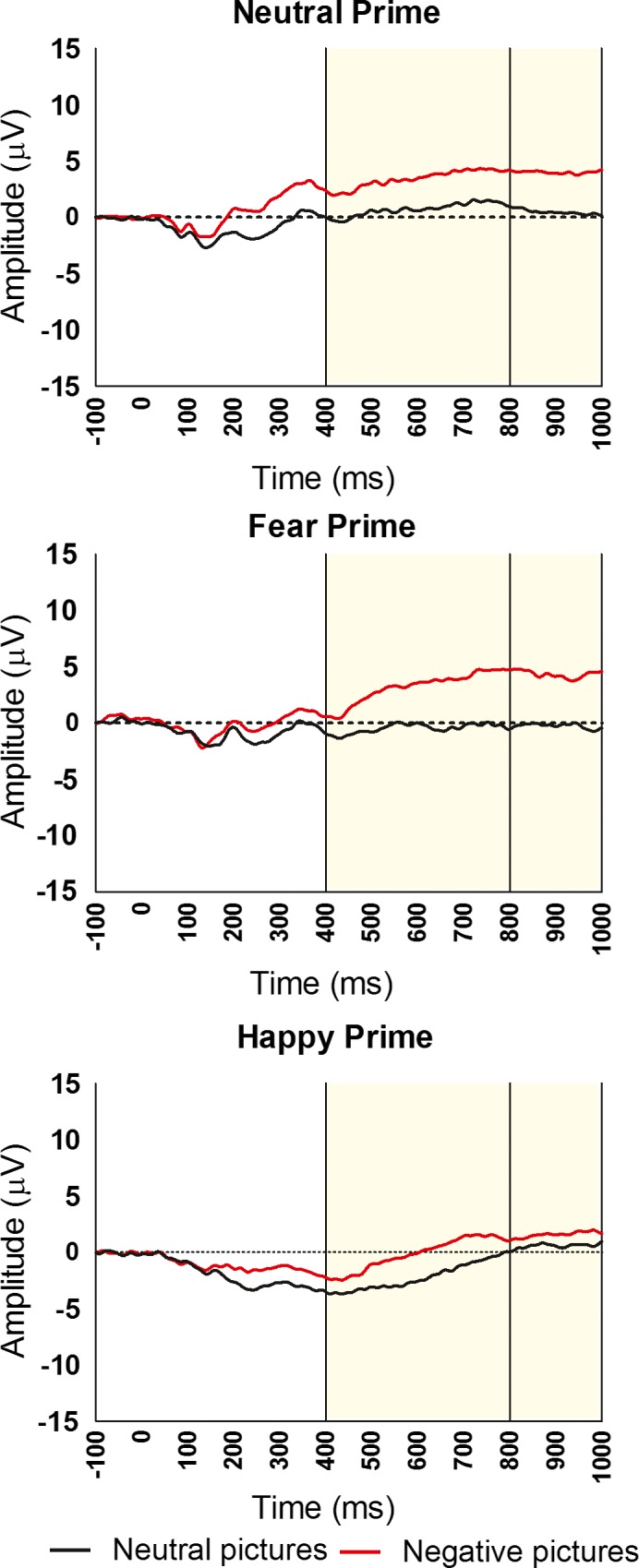
Visual LPP ERPs in response to emotionally negative (red) or emotionally neutral (black) images in the extrafoveal (top) and foveal (bottom) visual field, collapsed across priming condition (neutral, fearful, happy). Images were presented for 2000 ms. The LPP ERP was scored as the average activity from five sites (Cz, Pz, CPz, CP1, and CP2). *Y*‐axis represents voltage (µV), and *x*‐axis represents time (ms)

Results of the full model are presented in Table 1. In addition to a significant main effect for stimulus presentation location, the Condition × Latency and Location × Latency interaction effects were also significant (all *p*s < 0.05). Full interaction effects are shown in Figure 2 (by prime condition) and Figure 3 (by picture location).

**Table 1:** Three‐way mixed‐model ANOVA examining the effects of condition (happy, fear, neutral), LPP latency range (early, middle), and stimulus presentation location (foveal, extrafoveal) on LPP amplitude difference in response to negative vs. neutral pictures

	*df*	*F*	*p*	*η* ^2^_partial_
Condition	2, 46	1.14	0.33	0.05
Latency range	1, 46	1.36	0.25	0.03
Location	1, 46	12.19	<0.01	0.21**
Condition × latency	2, 46	3.43	0.04	0.13*
Condition × location	2, 46	0.52	0.60	0.02
Latency × location	1, 46	7.31	<0.01	0.14**
Condition × latency × location	2, 46	0.76	0.47	0.03

^*^ *p* < 0.05, ^**^ *p* < 0.01.

#### 3.3.1 Main effect of presentation location

Follow‐up analyses indicate a significantly larger difference in LPP amplitude between negative and neutral pictures for stimuli presented extrafoveally (*M*_diff_ = 8.6 µV, *SD* = 12.1) compared to foveally (*M*_diff_ = 1.1 µV, *SD* = 9.8), *t*(48) = 3.56, *p* < 0.01, *d* = 0.51. For stimuli presented foveally, there was no significant difference in LPP amplitude between negative (*M* = 2.5 µV, *SD* = 9.0) and neutral (*M* = 1.4, *SD* = 8.5) pictures, *t*(48) = 0.80, *p *> 0.05. For stimuli presented extrafoveally, however, the LPP amplitude was significantly larger for negative pictures (*M* = 6.4 µV, *SD* = 12.1) than for neutral pictures (*M* = −2.2 µV, *SD* = 8.7), *t*(48) = 4.98, *p* < 0.01.

#### 3.3.2 Location × Latency interaction

For stimuli presented extrafoveally, the difference in LPP amplitude between negative and neutral pictures was significantly larger in the middle window (*M*_diff_ = 10.2 µV, *SD* = 13.5) compared to the early window (*M*_diff_ = 7.0 µV, *SD* = 11.2), *t*(48) = 3.67, *p* < 0.01, *d* = 0.52. Conversely, for stimuli presented foveally, there was no significant difference in LPP amplitude between negative and neutral pictures in the middle window (*M*_diff_ = 0.3 µV, *SD* = 12.1) compared to the early window (*M*_diff_ = 1.9 µV, *SD* = 9.0), *t*(48) = 1.36, *p*>0.05.

#### 3.3.3 Condition × Latency interaction

For participants in the fear condition, the difference in LPP amplitude between negative and neutral pictures was significantly larger in the middle window (*M*_diff_ = 8.0 µV, *SD* = 11.2) compared to the early window (*M*_diff_ = 5.8 µV, *SD* = 10.2), *t*(15) = 2.38, *p* < 0.05, *d* = 0.60. No such difference in LPP amplitude between negative and neutral pictures was found between the early and middle windows for participants in either the happy condition, *t*(14) = 1.45, *p *> 0.05, or the neutral condition, *t*(17) = 1.29, *p *> 0.05. Additional analyses confirm that the LPP amplitude differentiates between negative and neutral pictures in both the early window (*p* < 0.05, *d* = 0.57) and middle window (*p* < 0.05, *d* = 0.61) for participants in the control condition and in the early window (*p* < 0.05, *d* = 0.70) for participants in the happy condition. However, for participants in the happy condition, the LPP amplitude no longer differentiated between negative and neutral pictures by the late window (*p *> 0.05, *d* = 0.37).

## 4 DISCUSSION

We aimed to investigate the effect of incidental emotion priming on attentional motivational systems as measured by the LPP ERP component for emotionally laden stimuli presented foveally and extrafoveally. In order to induce a target emotion, we presented each participant with one of three previously validated video clips to induce emotion changes: affectively neutral, affectively positive, or affectively negative. Following the incidental emotion manipulation, we presented affectively negative and affectively neutral visual stimuli, both foveally and extrafoveally, while simultaneously recording EEG ERP data.

Consistent with previous research, in our affectively neutral prime (i.e., control) condition, the LPP differentiated negative from neutrally valenced stimuli in both early (400–800 ms) and middle (800–1,000 ms) LPP latency ranges post stimulus. A similar moderately sized effect was found in our fear prime condition, although the size of the effect increased significantly from the early to the middle window. This suggests an increase in processing of affectively negative stimuli with increased latency while in an emotionally negative state. However, positive incidental affect seems to produce the opposite effect, where processing of negative emotional stimuli is no longer significantly differentiated from emotionally neutral stimuli by the middle LPP latency range. In other words, when in a happy state, processing of negative emotional stimuli is blunted as a function of latency. We interpret these results as support for the notion that, when in an affectively negative state, negative stimuli receive increasing attention whereas when in an affectively positive state, negative stimuli are dismissed from attention more quickly. A state of happiness, then, would appear to serve as an emotional prophylactic to subsequent negative stimuli.

The effect of a happy emotional state on the perception of negative stimuli may not be surprising, given prior research showing that an affectively positive state increases the accessibility of positive memories, positive events, and makes people more optimistic relative to negative affective states (Wegener & Petty, 1994). When individuals are in a positive incidental state, they tend to interpret events in a particularly positive light (“wearing rose‐colored glasses”) that coincides with their mood (Niedenthal, 1992; Niedenthal & Setterlund, 1994). Relatedly, a positive affective state can also influence an individual's attentional breadth, such that attention is narrowed for high approach motivation (positive) stimuli and broadened for low approach motivation (negative) stimuli (Gable & Harmon‐Jones, 2008; Harmon‐Jones & Gable, 2009). This may at least partly explain why the LPP failed to differentiate between negative and neutral stimuli in the after 800 ms epoch for participants in the happy prime condition. That is, when in an affectively positive state, emotional processing of negative stimuli was dismissed more readily (i.e., broadened attention) than when in a negative or neutral state, thus potentially providing some protective effect against deleterious effects of negative stimuli.

In addition to affective priming, we tested the effect of stimulus presentation location on the LPP response. It appears that, overall, negative images presented extrafoveally produced a more robust LPP ERP than did the same images when they were presented foveally. This relationship between LPP response and stimulus presentation location reveals an important distinction between foveal and extrafoveal emotion processing, where perception of emotional stimuli in the periphery shows heightened sensitivity as compared to perception in the foveal condition. This finding agrees with previous work showing accurate identification of emotional pictures up to 60º from a fixation point (D'Hondt, Szaffarczyk, Sequeira, & Boucart, 2016). Importantly, the processing of stimuli in the periphery is specific to emotional or salient stimuli. For example, people can detect and discriminate faces showing fear and disgust (threatening and potentially dangerous visual information) from neutral faces in the periphery (40º from center), but are unable to decimate the sex of the faces (Bayle et al., 2011). Previous research supports the notion that emotional stimuli can reliably be discriminated from neutral scenes when presented extrafoveally (Calvo, Rodriguez‐Chinea, & Fernandez‐Martin, 2015). Emotional stimuli take processing priority outside the focus of overt attention, thus decreasing processing for nonemotional extrafoveal stimuli (Calvo, Gutierrez‐Garcia, & Del Libano, 2015; Carretie, 2014). This parallels previous eye movement research exhibiting selective orienting toward extrafoveal emotional stimuli (Alpers, 2008; McSorley & van Reekum, 2013) and electrocortical research showing enhanced ERP amplitudes for emotional stimuli than neutral stimuli extrafoveally (De Cesarei, Codispoti & Schupp, 2009; Rigoulot et al., 2008). Our study builds on this work by suggesting that not only is emotional stimuli able to be detected in the peripheral visual system, but the neurophysiological processing of emotional stimuli in the periphery is more sensitive than when identical emotional stimuli are presented foveally (in terms of ERP LPP amplitude). Notably, however, one study found increased emotion processing for stimuli presented foveally but not extrafoveally (De Cesarei et al., 2009). Differences in study methodology could potentially explain the different findings between this study and our study. For example, picture on time was 24 ms in the De Cesarei study and 2000 ms in our study and the De Cesarei study included a distractor while our study did not.

One limitation of the present study is that we did not analyze the LPP after 1,000 ms (in order to measure the late LPP) due to an observed high amount of noise and eye artifact after this period. Although participants were told to blink only when the picture turned off (after 2000 ms) and were able to achieve this in the practice trials, it is possible that the combination of the video presentation, practice trials, and experimental protocol with a high number of trials resulted in difficulty maintaining stillness for longer than ~1,000 ms during the trials.

A second limitation is the potential confound introduced by different instructions provided for stimuli presented foveally vs. extrafoveally. Specifically, participants were instructed to rate the valence of the pictures when they were presented foveally but not when they were presented outside the center field of view (extrafoveally). Previous work has suggested that the LPP is relatively unaffected by various context manipulations showing similar ERP modulation for passively viewing pictures and making explicit evaluative ratings (Codispoti, Ferrari, Cesarei, & Cardinale, 2006; Cuthbert et al., 1995). However, it is possible that the difference in LPP that we attribute to presentation location is actually due to differences in task instruction and/or differences in attentional load subsequent to that instruction. We consider this to be unlikely, as results indicated a larger difference in LPP amplitude in the condition that is *absent* of overt instruction to rate the stimulus valence (i.e., in the extrafoveal condition). Were the different instructions to be the cause of the differences in LPP amplitude between conditions, we would expect to see results opposite of those presented here. Additionally, given the low attention demand of both conditions, it is also unlikely that this would be a significant confounding factor.

In sum, our data suggest that, when in a happy state, processing of negative emotional stimuli is blunted. In addition to supporting the perception of emotions as motivational system triggers, these results are consistent with the broaden‐and‐build theory of positive emotions (Fredrickson, 2004). Generally, this theory posits that the experience of positive emotions is associated with a broadening of both attentional focus and behavioral repertoire, which together allow for the building‐up of resources to successfully navigate physical and social environments. Previous research has suggested that this is partially accomplished by positive affect decreasing one's ability to focus on any single particular stimulus, thus forcing attention to expand (Rowe, Hirsch, & Anderson, 2007). Results of the current study are consistent with this research, in that participants in the happy prime condition appeared to dedicate fewer cognitive resources (as assessed via the LPP ERP) over a shorter period of time to processing negative stimuli. This is contrary to the general tendency for people to process negative stimuli more quickly and for longer periods of time (Carretie, Mercado, Tapia, & Hinjosa, 2001), a tendency that results in a negativity bias common to many psychopathologies. The fact that positive emotions may disrupt this negativity loop could prove valuable in the treatment of these disorders (Garland et al., 2010).

## 5 CONCLUSION

Incidental affective state alters emotional processing differentially for emotionally negative vs. emotionally neutral stimuli. In a neutral affective state, results reveal an increase in processing for negative stimuli as compared to neutral stimuli, an effect that is amplified by latency when in an affectively negative fearful state. Critically, in a positive affective state, emotional processing fails to differentiate negative and neutral stimuli by the middle LPP range (800–1,000 ms), suggesting negative stimuli are dismissed from attention more readily than when in either an affectively negative or affectively neutral state. This effect suggests a protective nature of incidental happiness against negative stimuli through expanding attentional mechanisms. Further, results show differential processing for emotionally negative and emotionally neutral stimuli in the foveal vs. extrafoveal stimulus locations, where perception of emotional stimuli extrafoveally shows heightened sensitivity to negative images. Overall, an interplay between incidental affective state, stimulus location, and LPP latency during emotional processing is affirmed.

## CONFLICT OF INTERESTS

The authors have no competing interests to declare.

## AUTHOR CONTRIBUTIONS

LDH, MF, and JLT designed the study; LDH performed the experiments; LDH, VGS, and JLT analyzed the data. LDH drafted the first version of the manuscript, and JLT, VGS and MF edited the manuscript. All authors discussed the results and interpretations.
